# Partial validation of a six-month high-fat diet and fructose-glucose drink combination as a mouse model of nonalcoholic fatty liver disease

**DOI:** 10.1007/s12020-024-03769-5

**Published:** 2024-03-20

**Authors:** Evangelia S. Makri, Konstantinos Xanthopoulos, Panagiotis Mavrommatis Parasidis, Eleftheria Makri, Spyros Pettas, Anastasia Tsingotjidou, Angeliki Cheva, Iris Ballaouri, Spyridon Gerou, Antonis Goulas, Stergios A. Polyzos

**Affiliations:** 1 First Laboratory of Pharmacology, School of Medicine, Aristotle University of Thessaloniki, Thessaloniki, Greece; 2 Laboratory of Pharmacology, School of Pharmacy, Aristotle University of Thessaloniki, Thessaloniki, Greece; 3 Institute of Applied Biosciences, Centre for Research and Technology, Thessaloniki, Greece; 4 Laboratory of Anatomy, Histology & Embryology, School of Veterinary Medicine, Aristotle University of Thessaloniki, Thessaloniki, Greece; 5 Department of Pathology, School of Medicine, Aristotle University of Thessaloniki, Thessaloniki, Greece; 6 Analysis Laboratories, Thessaloniki, Greece

**Keywords:** Fast food diet, Fibrosis, Mouse model, Nonalcoholic fatty liver disease, Nonalcoholic steatohepatitis, Steatosis

## Abstract

**Purpose:**

The need to investigate the pathogenesis and treatment of nonalcoholic fatty liver disease (NAFLD) has led to the development of multiple mouse models. The aim of this study was to validate a fast food diet (FFD) mouse model that is introduced as being close to the human disease.

**Methods:**

Eight to nine weeks old male and female C57BL/6 J mice were randomly allocated to a FFD group or to a chow diet (CD) group. Every four weeks, mice were weighed, and blood samples were collected for the measurement of glucose, alanine aminotransferase (ALT), aspartate aminotransferase (AST), triglycerides (TGs) and total cholesterol. After 25 weeks, mice were sacrificed, and liver tissue was histologically evaluated.

**Results:**

FFD mice gained more weight (*p* = 0.049) and presented a higher liver-to-body weight ratio (*p* < 0.001) compared to CD mice. FFD group presented with greater steatosis, hepatocellular ballooning and NAFLD activity score (NAS), whereas lobular inflammation and fibrosis were not significantly different compared to CD. When stratified by sex, NAS was different between FFD and CD groups in both male and female mice. Group by time interaction was significant for weight, ALT and cholesterol, but not for glucose, AST and TGs.

**Conclusion:**

FFD mice presented with morphologic and biochemical features of NAFLD and with greater hepatic steatosis, hepatocellular ballooning and NAS, but not lobular inflammation and fibrosis, compared to CD mice. These results only partly validate the FFD mouse model for NAFLD, at least for a 6-month feeding period.

## Introduction

Nonalcoholic fatty liver disease (NAFLD) affects millions of people and has become the most common chronic liver disease, at least in western societies [1]. Its rapidly increasing prevalence has been largely attributed to the rising rates of metabolic syndrome (MetS) [2]. Obesity, type 2 diabetes mellitus (T2DM) and insulin resistance (IR) constitute major contributors to the pathogenesis of NAFLD [2, 3]. The progression to fibrosis and even, cirrhosis and hepatocellular carcinoma (HCC) increase liver-related morbidity and mortality, but cardiovascular mortality and mortality due to extra-hepatic malignancies are also high in patients with NAFLD [4, 5].

Despite the high prevalence of NAFLD and its negative consequences, there is no licensed drug therapy, although multiple agents have been investigated or are under investigation, as either monotherapy or in combination [6–9], underlying the need for better understanding of the pathophysiology of the disease, which is multifactorial [10]. In this regard, appropriate preclinical animal models are vital to deeply comprehend the metabolic pathways leading to the development and progression of NAFLD, knowledge that is important for the discovery and investigation of specific therapeutic targets [11].

Although there are many recommended animal models of NAFLD, they are limited by the fact that some of them develop metabolic aberrations, such as IR and hepatic steatosis [12, 13], with no induction of hepatic inflammation and fibrosis, whereas others develop advanced histological features of NAFLD, such as hepatic inflammation and/or fibrosis, without inducing considerable IR and hepatic steatosis [11]. In this regard, several dietary mouse models of NAFLD have been introduced with various and partly conflicting results. In an effort to reproduce the full phenotypic spectrum of NAFLD, high fat diet (HFD) mouse models were developed using different fat content and fat source, as well as different composition in saturated and polysaturated fatty acids, leading to obesity and consequently NAFL [14]. However, in order to reproduce hepatic inflammation and fibrosis, HFD should be administered for a prolonged duration, which is time- and resource-consuming [15]. The high fat, high cholesterol (HFHC) diet was supported to reproduce NASH; however, the concentration of cholesterol in this diet is much higher than a human diet rich in cholesterol [14]. High-fat, high-fructose (HFHF) diet was introduced after the observation that western diet contains high fructose concentrations, including soft drinks [16]. Hepatic steatosis and inflammation were not quite different compared to HFD; high fibrosis markers were reportedly observed in HFHF diet, however, without histological confirmation [17].

All the above considered, a mouse model subjected to high-fat diet and fructose-glucose drink combination, which is similar to the human “fast food” diet [fast food diet (FFD) mouse model] has been suggested to develop the full phenotypic spectrum of NAFLD (i.e., IR, hepatic steatosis, inflammation and fibrosis) after six months on FFD [18]. Specifically, it was reported that mice on FFD develop early (within the first weeks) obesity, IR, diabetes and hepatic steatosis, and hepatic inflammation and fibrosis after four to six months [19]. These characteristics render the FFD model appealing, because the full spectrum of NAFLD is observed, which is induced by western diet, a major contributor of human NAFLD; thus, the model was introduced as mimicking the human disease to a considerable extent.

The main aim of this study was to validate the FFD mouse model in NAFLD, by evaluating the effect of a 6-month FFD on morphological, biochemical and histological parameters as compared with chow diet (CD) in male and female C57BL/6 J mice.

## Materials and methods

Male and female C57BL/6 J mice aged 8-9 weeks were purchased from and bred in the animal facility of the Laboratory of Anatomy, Histology & Embryology, School of Veterinary Medicine, Aristotle University of Thessaloniki, Greece. All mice were born and raised in the same laboratory, so no adaptation period was needed. They were housed in a specific pathogen-free controlled environment (20 ± 2^o^C temperature, 60% humidity, 12 h light/12 h dark cycle) and had *ad libitum* access to food and water. No environmental enrichment products were used. At baseline, the animals were randomly assigned into two groups, which received different diets for 25 weeks. The first group (FFD group) received sterilized FFD, producing 42% of energy from fat, and containing 0.21% per weight cholesterol (TD.88137, ssniff Spezialdiäten GmbH, Germany) and the second group (control group) received sterilized CD (V1534-703, ssniff Spezialdiäten GmbH, Germany), as previously reported [18, 20]. FFD mice received fructose (23.1 g/l) and glucose (18.9 g/l) (Sigma-Aldrich Chemie GmbH, Germany) in drinking water, as previously described [18]. The detailed compositions of both diets are summarized in Table 1. Four mice of the same sex and fed with the same diet were housed in each cage, which was appropriately cleaned once a week. Mean weekly food and water consumption were calculated based on the weekly difference between the food or water supplied and food or water remaining, respectively. The experiments were done following the Planning Research and Experimental Procedures on Animals: Recommendations for Excellence (PREPARE) guidelines and the manuscript was prepared according to the Animal Research: Reporting of In Vivo Experiments (ARRIVE) guidelines 2.0 (essential 10) [21, 22]. All animal procedures were carried out in accordance with the relevant National and European Union regulations and were approved by the General Directorate of Agriculture, Economy and Veterinary Medicine of the region of Central Macedonia, Greece, which reviewed and approved the study. The study was also approved by the Bioethics Committee, School of Medicine, Aristotle University of Thessaloniki, Greece.

**Table 1:** Composition of diets

Nutrients	Diet	
	FFD	CD
Protein (% weight)	17.3	19.0
Fat (% weight)	21.1	3.3
Carbohydrates (% weight)	48.7	40.5
Cholesterol (% weight)	0.21	0.014
Fiber (% weight)	5.0	4.9
Fructose in water (g/l)	23.1	0.0
Glucose in water (g/l)	18.9	0.0

*CD* chow diet, *FFD* fast food diet

Weight measurement and non-fasting blood sampling from the submandibular vein were performed at baseline and every four weeks until the end of the study. Blood obtained from mice was incubated for 60 min at room temperature and centrifuged at 13,000 × *g* for 15 min at 8 °C. Serum was collected and stored at −80 °C. Serum glucose, alanine aminotransferase (ALT), aspartate aminotransferase (AST), triglycerides (TGs) and total cholesterol were measured using standardized and automated procedures. Insulin levels were measured by using an ELISA kit (Cat. # EZRMI-13K, Millipore, Missouri, USA), according to the manufacturer’s instructions. At 6 months (25 weeks), mice were weighed, blood was collected from the submandibular vein and they, subsequently, anesthetized with isoflurane inhalation (Biovet, Greece) and euthanized. Liver tissue samples were collected and immediately transferred into 10% buffered formalin and embedded into paraffin. Sections of 4 μm thick per mice were stained with hematoxylin-eosin (H&E) and they were used for histopathological analysis. Picrosirius red staining was also used for fibrosis assessment. An experienced pathologist (AC), blinded to the origin of samples, evaluated hepatic steatosis, hepatocellular ballooning, lobular inflammation, NAFLD activity score (NAS) and hepatic fibrosis, according to the classification of NASH Clinical Research Network [23]. A second, independent reading of the samples was also performed (AG). Minimal disagreement was resolved by consensus in the presence of ESM and SAP.

### Statistical analysis

All animals were included in the analysis. Data are presented as mean ± standard deviation (SD) for continuous variables or number and/or frequencies for categorical variables. The Shapiro-Wilk test was used for the assessment of normal distribution. Homogeneity of variances between groups was tested with Levene’s test. The independent samples *t* test or the Mann–Whitney U test were used for between group comparisons of normally or non-normally distributed continuous variables, respectively. When the variance differed between the two groups (heterogeneity of variances) the Welch correction was applied. A repeated measures ANOVA (2 × 2) was performed to evaluate the food and water consumption, as well as the effect of different diets over time on various parameters. Sphericity correction applied to the output, when the sphericity assumption was violated (Mauchly’s test *p* value < 0.05); the Bonferroni *post-hoc* test was used to correct for multiple pairwise comparisons, in cases that repeated measures ANOVA provided a statistically significant p-value for trend within groups. The level of statistical significance was set at *p* value < 0.05. All statistical analyses were performed with R Studio version 1.4.1717 (the R Foundation for statistical computing, Vienna, Austria) and SPSS 29 for Windows (IBM Corp., Armonk, NY, USA).

## Results

### Comparison of FFD vs. CD

**Fig. 1:**
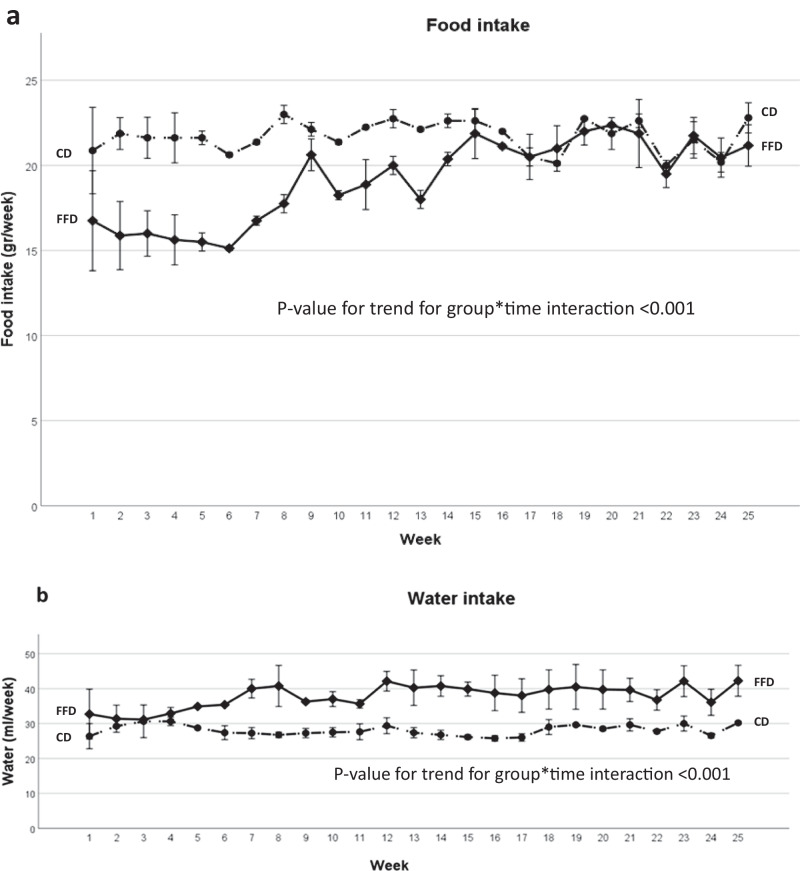
Food and water intake changes in C57BL/6 J mice on FFD (*n* = 8) and CD (*n* = 8) diet during a 6-month period. **a** Regarding food intake, there was a significant group by time interaction (*p* < 0.001), indicating that food intake was decreased in FFD mice at baseline compared to CD mice, but increased in FFD mice at the second half of the study, when similar food intake was observed between groups. **b** Regarding water intake, there was a significant group by time interaction (*p* < 0.001), indicating that water intake was increased in FFD mice compared to CD mice. Data are presented as mean ± standard deviation. Continue lines represent FFD group, discontinue line represent CD group. Error bars represent standard deviation. CD chow diet, FFD fast food diet

Sixteen C57BL/6 J mice were used for the needs of this study. Eight mice (4 male) received FFD and 8 mice (4 male) received CD. Food and water intake were measured at weekly intervals (Fig. 1). There was significant group by time interaction on food and water (Table 2). Specifically, the quantity of food intake was initially significantly lower in FFD mice compared to the CD mice (p for trend <0.001); but was subsequently increased to control (CD) levels during the second half of the study (Fig. 1a). Thus, the food consumption was increased in FFD mice overtime, a difference that met the level of statistical significance, as compared to week 1, at the last 3 weeks of the study (week 23: *p* < 0.001, week 24: *p* = 0.02, week 25: *p* < 0.001). On the contrary, food consumption in CD mice was stable throughout the study and not significantly different compared to week 1. FFD mice consumed significantly more water than CD mice, from week 4 and onwards (Fig. 1b). Water intake in CD mice was stable during the study, similarly to food intake.

**Table 2:** Comparative baseline and endpoint data for both groups for parameters with serial measurements

Variable		FFD		CD		*p* value for group*time interaction trend
		Baseline	Endpoint (week 25)	Baseline	Endpoint (week 25)	
Body Weight (g)	total	18.1 ± 3.1	31.3 ± 5.8	19.0 ± 4.6	26.0 ± 3.8	<0.001
	male	20.9 ± 0.4	36.0 ± 3.6	23.2 ± 1.0	29.5 ± 0.6	<0.001
	female	15.2 ± 0.5	26.5 ± 2.1	14.8 ± 1.1	22.5 ± 1.0	<0.001
Food intake (g/week)	total	16.8 ± 2.9	21.2 ± 1.2	20.9 ± 2.5	22.8 ± 0.9	<0.001
	male	19.5 ± 0.0	22.3 ± 0.0	23.3 ± 0.0	23.6 ± 0.0	<0.001
	female	14.0 ± 0.0	20.0 ± 0.0	18.5 ± 0.0	22.0 ± 0.0	<0.001
Water intake (ml/week)	total	32.7 ± 7.2	42.2 ± 4.4	26.4 ± 3.6	30.2 ± 0.5	<0.001
	male	39.4 ± 0.0	46.4 ± 0.0	29.8 ± 0.0	30.6 ± 0.0	<0.001
	female	26.0 ± 0.0	38.1 ± 0.0	23.0 ± 0.0	29.8 ± 0.0	<0.001
Glucose (mg/dl)	total	101.3 ± 21.5	95.4 ± 18.9	101.4 ± 43.9	69.1 ± 18.2	0.32
	male	109.8 ± 27.8	97.0 ± 24.0	132.0 ± 0.0	67.5 ± 25.4	0.14
	female	92.8 ± 10.9	93.8 ± 15.8	70.8 ± 44.8	70.8 ± 11.0	0.47
AST (IU/L)	total	94.9 ± 25.8	101.1 ± 37.5	86.9 ± 18.1	66.8 ± 11.1	0.12
	male	82.0 ± 12.5	79.8 ± 21.1	75.8 ± 17.3	63.3 ± 14.4	0.43
	female	107.8 ± 30.8	122.5 ± 40.3	98.0 ± 11.4	70.3 ± 6.9	0.19
ALT (IU/L)	total	36.9 ± 15.6	66.0 ± 17.1	45.6 ± 17.0	46.3 ± 8.4	0.001
	male	32.3 ± 13.7	58.0 ± 8.4	46.0 ± 23.6	44.3 ± 10.2	0.01
	female	41.5 ± 18.0	74.0 ± 21.0	45.3 ± 10.9	48.3 ± 7.0	0.04
TGs (mg/dl)	total	103.0 ± 20.6	75.5 ± 31.0	115.0 ± 44.7	111.0 ± 33.7	0.53
	male	120.0 ± 8.5	79.2 ± 41.7	156.0 ± 7.1	136.0 ± 32.5	0.10
	female	85.8 ± 12.1	71.8 ± 21.5	74.5 ± 12.8	86.8 ± 3.4	0.06
Cholesterol (mg/dl)	total	66.1 ± 11.6	160.8 ± 53.3	66.3 ± 13.4	80.3 ± 16.4	<0.001
	male	72.3 ± 11.0	194.5 ± 56.9	73.0 ± 14.9	94.8 ± 5.4	<0.001
	female	60.0 ± 9.8	127.0 ± 18.7	59.5 ± 8.7	65.8 ± 6.3	0.01

Data are presented as mean ± standard deviation

*ALT* alanine aminotransferase, *AST* aspartate aminotransferase, *CD* chow diet, *FFD* fast food diet, *TGs* triglycerides

**Fig. 2:**
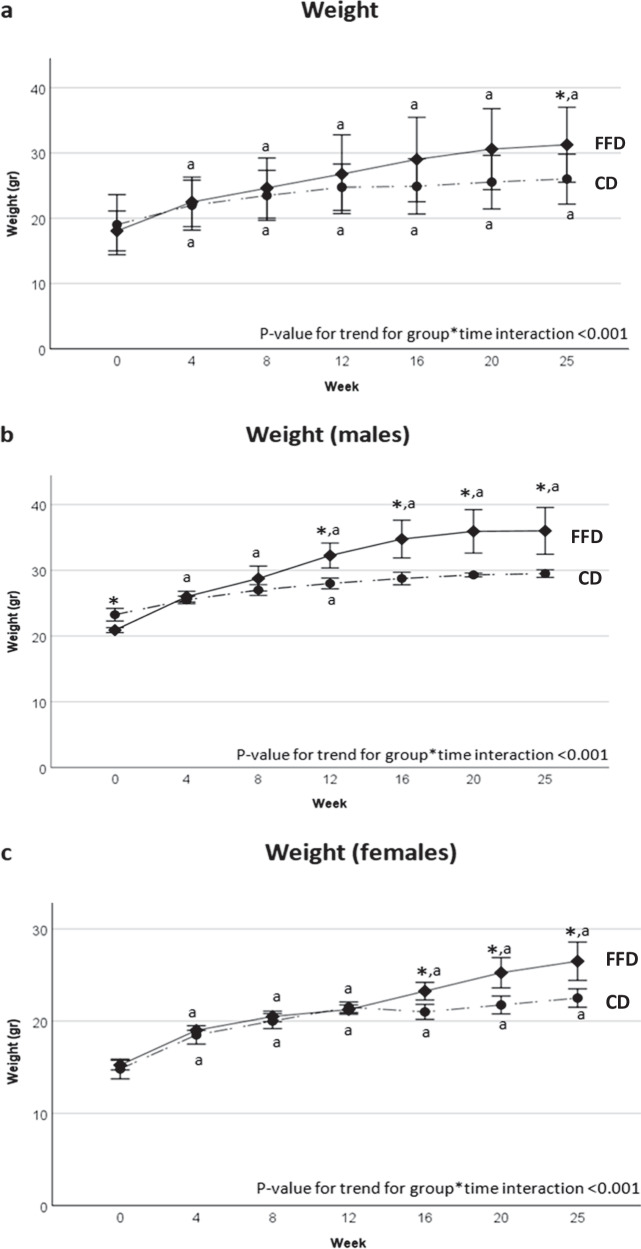
Body weight changes in C57BL/6 J mice on FFD and CD diet during a 6-month period. **a** When all mice were considered (FFD: n = 8; CD: n = 8), there was a significant group by time interaction (*p* < 0.001), indicating that FFD mice gained more weight than CD mice overtime. **b** In male mice (FFD: n = 4; CD: n = 4), there was a significant group by time interaction (*p* < 0.001), indicating that FFD male mice gained more weight than CD male mice overtime. **c** In female mice (FFD: n = 4; CD: n = 4), there was a significant group by time interaction (*p* < 0.001), indicating that FFD female mice gained more weight than CD female mice overtime. Data are presented as mean ± standard deviation. Continue lines represent FFD group, discontinue line represent CD group. Error bars represent standard deviation. CD chow diet, FFD fast food diet. *p < 0.05 compared to CD at the same time point (between group comparisons).^a^p < 0.05 compared to baseline (within group comparisons)

At the end of the 25-week study period, FFD mice had gained significantly more weight (mean weight gain approximately 13 g) compared to CD mice (mean weight gain approximately 7 g). There was a statistically significant group by time interaction for body weight (*p* < 0.001; Table 2). Specifically, mouse weight significantly increased within both groups from week 4 onwards with the trend being higher in the FFD group; however, the between group difference was statistically significant only at the endpoint (Fig. 2a, Table 3).

**Table 3:** Between group comparative morphologic, biochemical and histological parameters at the endpoint and in both sexes

Variable		FFD	CD	*p* value
Body weight (g)	total	31.3 ± 5.8	26.0 ± 3.8	0.049
	male	36.0 ± 3.6	29.5 ± 0.6	0.01
	female	26.5 ± 2.1	22.5 ± 1.0	0.01
Liver weight (g)	total	1.6 ± 0.3	1.0 ± 0.1	<0.001
	male	1.9 ± 0.3	1.2 ± 0.0	0.11
	female	1.4 ± 0.2	0.9 ± 0.1	<0.001
Liver-to-body weight ratio (%)	total	5.2 ± 0.2	4.06 ± 0.2	<0.001
	male	5.2 ± 0.3	3.9 ± 0.0	0.04
	female	5.3 ± 0.2	4.1 ± 0.2	<0.001
Glucose (mg/dl)	total	95.4 ± 18.9	69.1 ± 18.2	0.01
	male	97.0 ± 24.0	67.5 ± 25.4	0.14
	female	93.8 ± 15.8	70.8 ± 11.0	0.054
AST (IU/l)	total	101.1 ± 37.5	66.8 ± 11.1	0.03
	male	79.8 ± 21.1	63.3 ± 14.4	0.24
	female	122.5 ± 40.3	70.3 ± 6.9	0.04
ALT (IU/l)	total	66.0 ± 17.1	46.3 ± 8.4	0.01
	male	58.0 ± 8.4	44.3 ± 10.2	0.08
	female	74.0 ± 21.0	48.3 ± 7.0	0.059
TGs (mg/dl)	total	75.5 ± 31.0	111.0 ± 33.7	0.045
	male	79.2 ± 41.7	136.0 ± 32.5	0.08
	female	71.8 ± 21.5	86.8 ± 3.4	0.22
Total cholesterol (mg/dl)	total	160.8 ± 53.3	80.3 ± 16.4	0.001
	male	194.5 ± 56.9	94.8 ± 5.4	0.01
	female	127.0 ± 18.7	65.8 ± 6.3	<0.001
Insulin (ng/ml)	total	0.7 ± 0.3	0.3 ± 0.2	0.01
	male	0.8 ± 0.4	0.4 ± 0.2	0.11
	female	0.5 ± 0.1	0.2 ± 0.1	<0.001
Steatosis grade	total	1.0 ± 1.1	0.0 ± 0.0	0.03
	male	1.5 ± 1.3	0.0 ± 0.0	0.14
	female	0.5 ± 0.58	0.0 ± 0.0	0.43
Hepatocellular ballooning	total	1.8 ± 0.5	0.3 ± 0.7	0.002
	male	1.8 ± 0.5	0.5 ± 1.0	0.14
	female	1.8 ± 0.5	0.0 ± 0.0	0.03
Lobular inflammation	total	1.1 ± 0.4	0.6 ± 0.5	0.12
	male	1.3 ± 0.5	0.8 ± 0.5	0.57
	female	1.0 ± 0.0	0.5 ± 0.6	0.43
NAS	total	3.9 ± 1.5	0.9 ± 1.0	<0.001
	male	4.5 ± 1.7	1.3 ± 1.3	0.02
	female	3.3 ± 1.0	0.5 ± 0.6	0.03
Hepatic fibrosis	total	0.6 ± 0.5	0.1 ± 0.4	0.12
	male	0.8 ± 0.5	0.3 ± 0.5	0.49
	female	0.5 ± 0.6	0.0 ± 0.0	0.43

Values are presented as mean ± standard deviation

*ALT* alanine aminotransferase, *AST* aspartate aminotransferase, *CD* chow diet, *FFD* fast food diet, *NAFLD* nonalcoholic fatty liver disease, *NAS* NAFLD activity score, *TGs* triglycerides

Regarding biochemical tests, ALT, AST, glucose, insulin and total cholesterol were significantly higher in FFD compared to CD mice at the endpoint, whereas TGs were higher in CD compared to FFD mice (Table 3). Endpoint ALT and cholesterol were also elevated compared to baseline within the FFD group (*p* = 0.01 and p = 0.002, respectively), but not within the CD group, while TGs were decreased within FFD (*p* = 0.04) (Table 2). Glucose and AST were not statistically different within either group. Regarding the group by time interaction, there was a significant trend for ALT and total cholesterol, but not for glucose, AST and TGs (Table 2).

Endpoint liver weight and liver-to-body weight ratio were significantly higher in the FFD compared to the CD group, indicating hepatomegaly in the FFD group (Table 3). Regarding histological parameters, significantly greater steatosis and hepatocellular ballooning were observed in the FFD compared to the CD group (Table 3, Supplementary Figure 1). In the FFD group, steatosis and hepatocellular ballooning were graded as 0-3 and 1-2, respectively, according to the classification of NASH Clinical Research Network [23]; on the contrary, steatosis and hepatocellular ballooning was scarcely observed in CD mice. The differences in lobular inflammation and hepatic fibrosis were not significant between the FFD and CD group, although early fibrosis (F1) was practically observed only in some (*n* = 5/8) FFD mice (Table 3). NAS was significantly higher in FFD than CD mice, apparently driven by the between group differences in hepatic steatosis and hepatocellular ballooning, but not lobular inflammation.

### Comparison of FFD vs. CD within subgroups of different sex

Regarding food and water intake, there was significant group by time interaction within both sexes (Table 2). However, a significant difference in food and water intake between male and female mice was also observed (group by time by sex interaction for food intake: p < 0.001; group by time by sex interaction for water consumption: p < 0.001). This was mainly attributed to the difference in food and water consumption observed between the male and female CD but not between the male and female FFD mice; specifically, food and water intake remained grossly stable in male, but increased in female CD mice, whereas food and water consumption increased in both male and female mice in FFD group (Table 2).

Body weight increased more in both male and female mice fed on FFD compared to CD: there was a significant group by time interaction for both sexes (Fig. 2b, c, Table 2). More specifically, male mice on FFD achieved significantly greater weight than male CD mice, from week 12 and onwards. Within group comparisons showed that body weight was significantly increased at all time points compared to baseline, mainly within the FFD male subgroup (Fig. 2b). Concerning female mice, body weight gain was again greater in FFD mice compared to CD mice, but only after week 16 (Fig. 2c). Within groups comparisons showed that body weight was significantly increased at all time points compared to its baseline value in both female subgroups (Fig. 2c).

Biochemical parameters showed similar trends in the sex-defined subgroups as in the sum of the mice. ALT and total cholesterol, but not glucose, AST and TGs, showed a significant group by time interaction, in both sexes (Table 2). Between group comparisons at the endpoint showed that total cholesterol was higher in the FFD than in the CD subgroup, irrespective of sex; ALT was marginally different between the FFD and CD subgroups; AST was significantly different between FFD and CD only in the subgroup of female mice; glucose and TGs were not significantly different between FFD and CD in either sex-defined subgroup (Table 3). Insulin levels were significantly higher in the FFD female group compared to CD, but no difference was observed between male groups (Table 3).

**Fig. 3:**
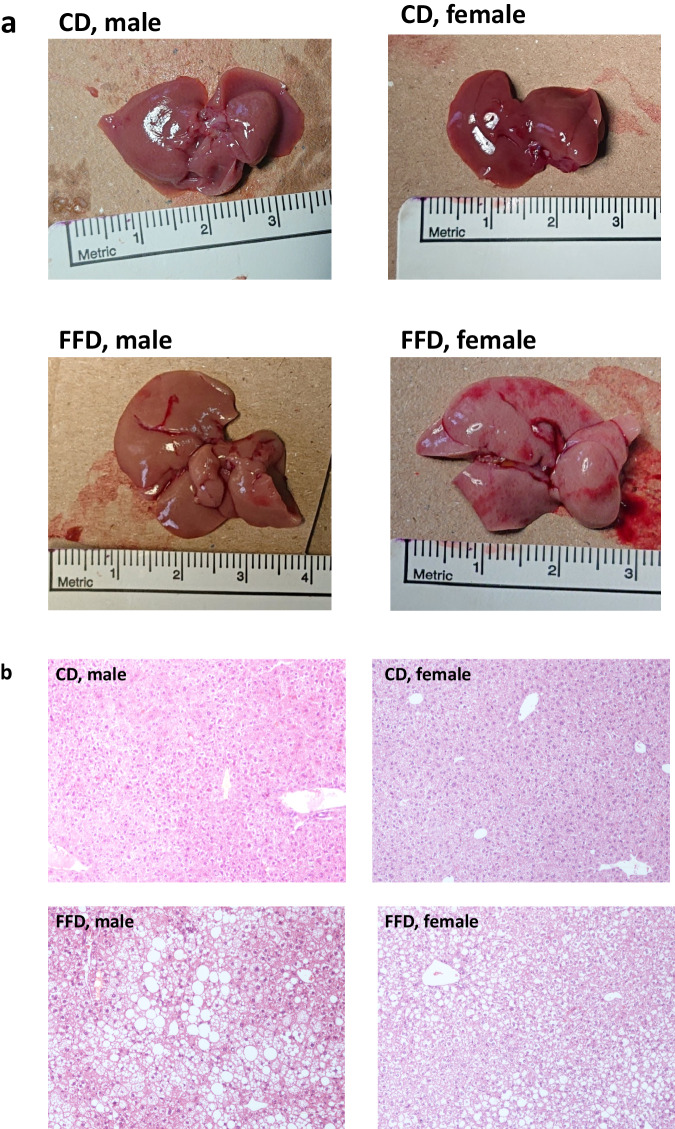
Histological evaluation of liver morphology and hepatic steatosis at week 25. **a** Dissected livers of FFD mice presented more yellowish than the livers of CD mice, which is characteristic of hepatic steatosis. **b** Two representative cases of hepatic steatosis in male and female mice on FFD compared with normal histology observed in a male and female mouse on CD (hematoxylin & eosin staining, magnification ×100). CD chow diet, FFD fast food diet

**Fig. 4:**
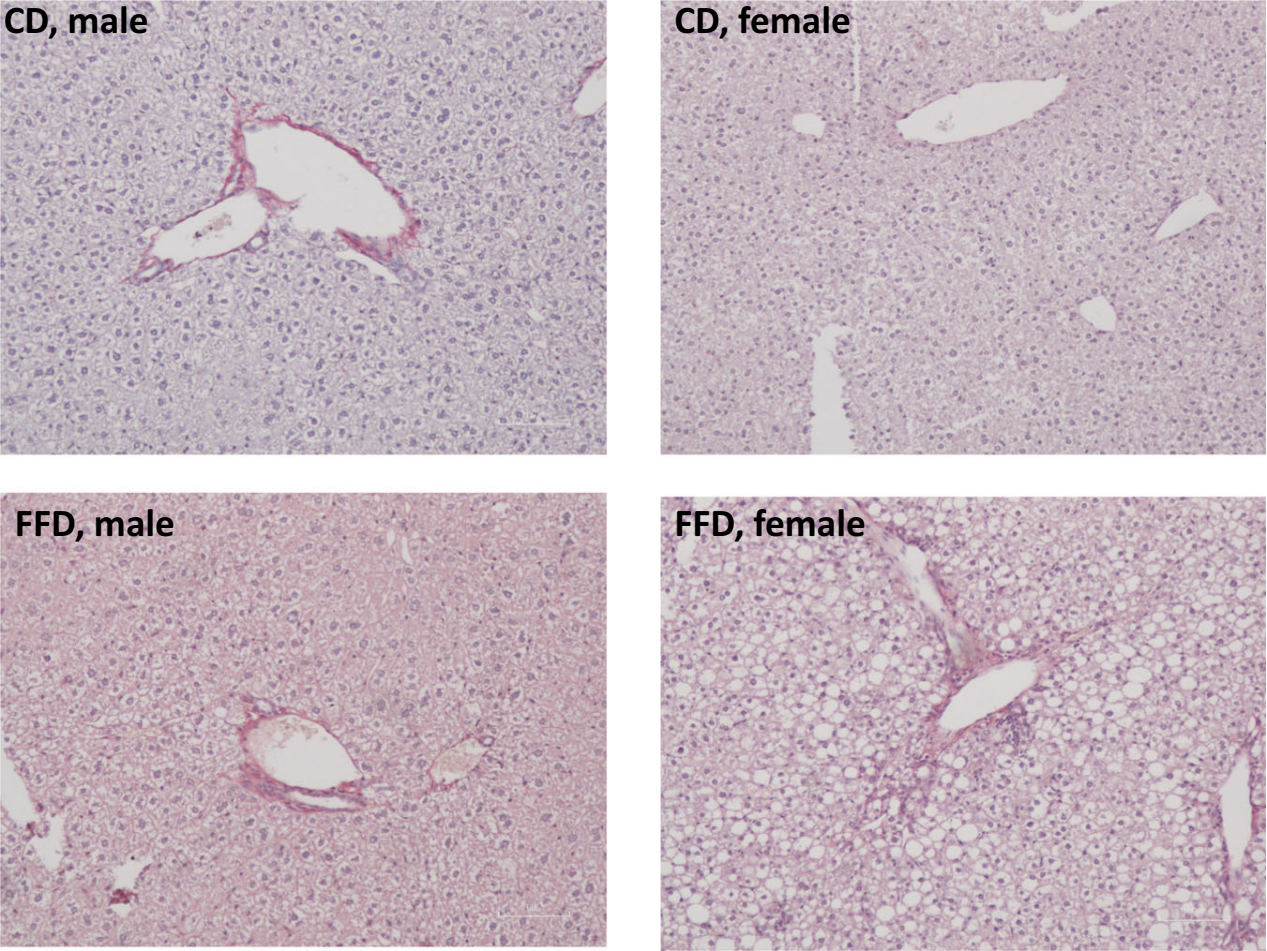
Histological evaluation of hepatic fibrosis at week 25. Two representative microphotographs of hepatic fibrosis in male and female mice on FFD compared with normal histology observed in a male and female mouse on CD; collagen fibers, are not detected in CD mice, whereas some collagen reactivity is observed in FFD mice (picrosirius red staining, magnification ×100). However, the differences in hepatic fibrosis were not statistically significant between the FFD and CD mice. CD chow diet, FFD fast food diet

Male mice did not show between group statistical differences in liver weight, while the liver-to-body weight ratio was significantly increased in the FFD compared to the CD subgroup. In female mice, liver weight and liver-to-body weight ratio were higher in FFD compared to CD mice (Table 3). Regarding histological features, despite numerical superiority in FFD than CD subgroups in all histological parameters, the level of statistical significance between FFD and CD subgroups was reached for NAS in both male and female mice (Fig. 3, Fig. 4, Table 3) and for hepatocellular ballooning in female mice. The higher values of steatosis, lobular inflammation and fibrosis observed in FFD compared to CD mice were not statistically significant. (Table 3).

## Discussion

In this study, we tried to validate and better characterize a previously introduced animal model (FFD mice), reported to simulate the whole spectrum of human NAFLD phenotype [18, 19]. In that model a diet high in saturated fats, cholesterol and fructose, resembling the composition of human “fast food” diet is used in order to simulate nutrition-induced NAFLD. The model was introduced as reproducing not only steatosis, but also NASH and hepatic fibrosis [18]. Its importance is supported by the fact that westernized diet is considered a major pathogenic contributor to the development and progression of NAFLD [2, 24]. Another key point of this model is the addition of fructose, a monosaccharide that is abundant in many processed foods, which is considered to be a major dietary contributor to the pathogenesis of NAFLD, by increasing de novo lipogenesis in hepatocytes, and upregulating tumor necrosis factor (TNF)-α, independently of insulin stimulation [25, 26]. Last but not least, the FFD mouse model is easily reproducible.

Although there are several animal models of NAFLD, in some of them NAFLD is developed without the reproduction of relevant metabolic parameters (e.g., IR, obesity, T2DM), whereas the full phenotypic histological range of NAFLD (steatosis, NASH, fibrosis and/or cirrhosis) is not developed in others. For example, the choline-deficient model leads to NASH and possible fibrosis, but it is also characterized by weight loss and low insulin levels, findings that do not characterize human NAFLD, which is accompanied by abdominal adiposity and high insulin levels (IR) [27]. Another well-known model, the *ob/ob* (leptin-deficient) mouse model, can hardly develop hepatic fibrosis when fed on CD [28, 29]. On the other hand, the FFD mouse was introduced to reproduce major hallmarks of human NAFLD, both metabolic (IR, obesity and T2DM) and histological, as opposed to other HFD models [18, 30]. As mentioned above, other relevant models, such as HFD, HFHC, HFHF, did not seem to reproduce the full spectrum of NAFLD, and/or they do not fully resemble human western diet [14, 17].

In this study, mice fed FFD developed hepatomegaly with greater liver-to-body weight ratio, as well as greater hepatic steatosis, hepatocellular ballooning and NAS than mice fed CD. Mean hepatocellular ballooning in FFD mice was similar to that of the introductory study of FFD mouse model (1.8 vs. 1.6, respectively) [18], but mean hepatic steatosis was lower in our study (1.0 vs. 2.7, respectively). Indeed, the validation of the development of hepatocellular ballooning, which is a hallmark of the intrahepatic inflammation, in FFD model is important, because it is difficult to develop in rodent models of NAFLD [31]. Furthermore, there was no difference between FFD and CD group in lobular inflammation, in contrast to the introductory study (mean values 1.1 vs. 2.5, respectively) [18], as well as in hepatic fibrosis (0.6 vs. 1.6, respectively). Of note, in our study, hepatic steatosis was not observed in any of the CD mice, while early fibrosis (F1) was observed in only five out of eight FFD mice. Six years after their introductory article [18], the same group conducted a longer-term study (36 weeks) with FFD and CD mice, housing two mice per cage [19], a condition slightly different from the introductory study, in which one mouse was housed per cage [18]. It is also important to highlight that, in their second study, only male mice were used, whereas female and male mice were used in equal numbers in the introductory study. In the second study, steatosis was greater in FFD mice from the first week, while inflammation was greater in FFD group in week 24 and onwards, and fibrosis was greater in FFD group from week 16 and onwards [19]. Contrary to the findings of the introductory study [18] and to those of our study, no evidence of hepatocellular ballooning was reported in that second study [19]. These findings underline the difficulty in reproducing characteristics of NASH and fibrosis in dietary animal models.

Other authors have published on similar dietary models of NAFLD, with varying results. Abe et al. showed greater steatosis and NAS, but they did not observe different inflammation score or hepatocellular ballooning even at week 30 in high-fat, high-fructose and high-cholesterol fed male mice; in their study, fibrosis was greater compared to CD mice at weeks 18 and 30, but not at week 24 [32]. Kristiansen et al. induced greater steatosis, inflammation, NAS and fibrosis, but not hepatocellular ballooning at week 26, in their high-fat, high-fructose and high-cholesterol model [28]. It is important to note, however, that in the studies of Abe and Kristiansen [28, 32], 2% w/w cholesterol was used, which represents a highly excessive amount, while we used 0.2% cholesterol, as corrected by Charlton et al. in 2015 [20]. This constitutes an important correction, since 0.2% rather than 2% simulates better the percentage of cholesterol in human western-type diet. A very high dietary cholesterol intake (1–2%) would radically surpass the daily recommended cholesterol consumption in humans and is, thus, far from a realistic intake [11]. On the other hand, higher dietary cholesterol intake in murine models may be required to reproduce NASH, and especially fibrosis, due to differences in metabolism between humans and mice, as the latter have a limited ability to metabolize cholesterol in the gut [33, 34]. In line with this, a FFD mouse model on high-fat, high-fructose diet and cholesterol developed greater NAS compared to the low-fat control group only when it was fed 2%, but not 0.2% cholesterol [35]. Kim et al. who used FFD with 0.2% cholesterol and approximately 22 g high-fructose corn syrup in the water, reported the development of steatosis and lobular inflammation without, however, evidence of fibrosis [36]. Based on the above, it seems that FFD may be provided for longer than 6 months to reproduce the full histological spectrum of NAFLD in a mouse model; this, however, remains to be investigated. All in all, it is important to note that different studies have used different types of FFD diets, and that creates serious comparability issues when it comes to their results. While it seems that the FFD mouse model resembles human disease to a considerable extent, it does need standardization in order to facilitate the interpretation of results and their comparability to those of other studies.

Regarding the metabolic features of NAFLD, we confirmed that FFD mice gained more weight and had higher glucose, cholesterol, insulin and liver function tests at the endpoint compared to CD mice. Compared with the introductory study by Charlton et al., in which the mean weight of mice at baseline was about 30 g, reaching 44.9 g on week 25 [18], our FFD mice had a mean weigh of 18.1 g at baseline, reaching 31.3 g on week 25 (Table 2); the histological differences observed between these studies may partly be attributed to the difference in baseline weight, while weight gain was similar. Charlton et al. showed higher circulating glucose, AST and cholesterol in FFD group in their introductory study [18], however, the elevations in glucose and AST were not different in their second study at week 24 [19]. Other studies using FFD reported various outcomes concerning biochemical parameters. A steady finding seems to be total cholesterol, which was shown to be increased after FFD in almost all relevant studies [28, 32, 37]. Some [17, 32, 37], but not all studies [28], agree with our results on ALT levels. As far as glucose levels are concerned, the results of different studies showed great variability, showing higher glucose levels in FFD mice at some time points, but not at the endpoints of 24-30 weeks in some studies [17, 28, 32]. TGs were higher in the CD group compared to the FFD group at the endpoint. This seemingly paradoxical finding was also observed by other authors [19, 32]. Hyperinsulinemia and IR were also observed in other NAFLD models, as well as in NASH patients even in the absence of obesity [18, 36, 38]. These results further emphasize the need for standardization of the FFD model, so as to lead to more reproducible results in relevant metabolic parameters, in line with the histological ones.

Lack of physical activity and a sedentary lifestyle promote the development and progression of NAFLD [39]. To enhance sedentary pattern, Charlton et al. used individual cages for mice (one mouse per cage) in the introductory study [18] and two mice per cage in their second study [19], whereas we placed four mice per cage, so as to restrict excessive movements without limiting the interaction with other mice and preventing stress associated with single housing. However, it would be useful to compare the size of the cages and the exact mean space given for each mouse, because it may probably account, at least partly, for the observed differences in different studies [18, 19].

Food consumption was greater in CD mice and stable during the study in this group, whereas FFD mice increased food consumption after the middle of the study, when they gained weight, more than the CD mice. On the contrary, water consumption was greater in FFD group, probably owing to the addition of fructose and glucose in the water, which may have made it more palatable to the mice. Caloric intake was not recorded in the introductory study [18]. The amount of food and water consumption is commonly not reported in animal studies, despite being critical variables, especially in studies in which nutrition and subsequent weight changes may have affected metabolic parameters or dosage of medications. Other C57BL/6 mice studies also reported lower food consumption in HFD group than in CD group [32, 40]. Interestingly, it has been indicated that sucrose-sweetened soft drinks reduce energy intake, thus increasing the possibility of a positive energy balance [41]. Of note, there is evidence demonstrating a negative effect of free fructose consumption on central appetite [42]. These data may explain the lower food intake at the first half of the study in FFD mice.

It should be highlighted that, with the exception of the introductory study of Charlton et al. [18], all relevant FFD studies used exclusively male mice. In our study, mice of both sexes were used to show differences in food and water consumption between male and female mice, which may partly explain a later weight gain in female compared to male mice (Fig. 2b, c, Table 2). This may have led to minimal biochemical and histological differences between subgroups of male and female mice (Table 3), which, however, could not explain the male predominance in the relevant studies. In humans, NAFLD prevalence is lower in premenopausal women compared to men of similar age, but after menopause, the prevalence of NAFLD becomes almost similar between men and women; this may be related to metabolic alterations following the abrupt decrease in estrogens in women after menopause [43–45]. These disparities between sexes have been demonstrated in some murine models confirming that female animals present with milder hepatic steatosis, inflammation and even fibrosis than male ones [46, 47]. The majority of preclinical studies use male mice, thus female mice are understudied, which may be misleading when the results of studies from male mice are extrapolated to female ones. In this regard, the effect of FFD on NAFLD in a longer-term, i.e., after menopause of female mice could be important. Alternatively, one could study the effect of FFD diet on NAFLD in ovariectomized female mice. We should also highlight that the dissemination of the negative results of studies is important to guide other researchers in the field in a global basis, so as not to invest their effort, time and resources on topics having previously provided negative results.

Last but not least, we could not overlook the recent change in the nomenclature of NAFLD to metabolic dysfunction-associated steatotic liver disease (MASLD) proposed by a multinational consensus [48]. This proposal is supported by the ascertainment that the use of “nonalcoholic” overemphasizes the absence of alcohol, whereas at the same time it underemphasizes the significance of multiple metabolic factors contributing to the development and progression of NAFLD [49]. The diagnosis of MASLD is based on definite criteria, i.e., hepatic steatosis along with at least one out of five metabolic risk factors, which may better reflect the pathophysiology of this highly heterogeneous disease and may facilitate research in the field with future clinical implications. However, in our opinion, the terms of NAFLD and MASLD, as well as NASH and metabolic dysfunction-associated steatohepatitis (MASH), may not be used invariably, since they are similar but not synonymous entities, until more evidence clarifies this issue.

This study has certain strengths and limitations. To our knowledge, this is the first study aiming to validate the model introduced by Charlton et al. [18], a western-type diet with 0.2% cholesterol and fructose in C57BL/6 J mice that simulates the fast food human diet, using both male and female mice. However, certain limitations of this study are: (1) the sample size was relatively small, especially for the analyses of subgroups stratified by sex (four mice per group), so the relevant findings should be cautiously interpreted, because they may be underpowered, e.g., findings on lobular inflammation; (2) we could not house one mouse per cage due to the induced stress that may have affected the outcomes of the study; (3) non-fasting blood sampling may have distorted blood tests results, e.g., glucose and TGs.

In conclusion, this study did not fully reproduce all elements of NAFLD in FFD male and female mice: hepatic steatosis was developed to a lower grade, and lobular inflammation and fibrosis were not developed so extensively as in the introductory study [18]. However, we could not overlook the development of hepatocellular ballooning and the association of histological evolution of NASH with morphological and metabolic changes characteristic of NAFLD. A longer duration of the study might have led to greater steatosis, lobular inflammation and fibrosis, but it remains to be shown. The FFD mouse model hold hope for the future of NAFLD research, but, until then, the diet should be standardized and some relevant issues (e.g., mice per cage, the selection of male and/or female mice) should be clearly determined.

### Supplementary information

Supplementary Figure

## Data Availability

The dataset generated during this study is available by the corresponding author on reasonable request.
