# Supplementary figures and images for: Partial validation of a six-month high-fat diet and fructose-glucose drink combination as a mouse model of nonalcoholic fatty liver disease
Source: Endocrine. 2024 Mar 20;85(2):704–16. doi: 10.1007/s12020-024-03769-5 (PMC11291610; doi:10.1007/s12020-024-03769-5)

## Slide 1
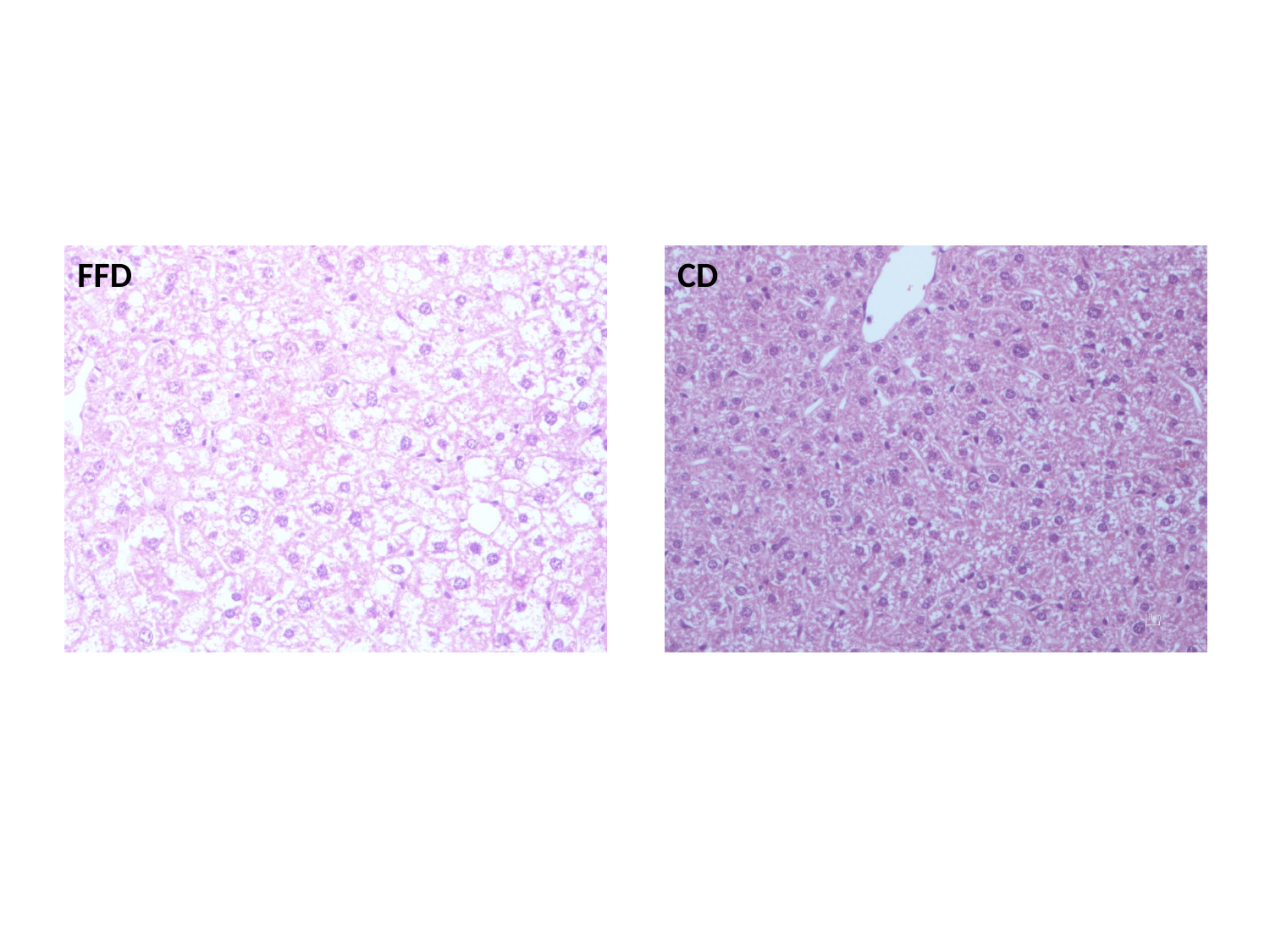

FFD
CD

Supplement: Supplementary file 1 — Supplementary Figure [file 12020_2024_3769_MOESM1_ESM.pptx]
